# A coherent mathematical characterization of isotope trace extraction, isotopic envelope extraction, and LC-MS correspondence

**DOI:** 10.1186/1471-2105-16-S7-S1

**Published:** 2015-04-23

**Authors:** Rob Smith, John T Prince, Dan Ventura

**Affiliations:** 1 Department of Computer Science, University of Montana, 59812 Missoula, USA; 2 Department of Chemistry, Brigham Young University, 84606 Provo, USA; 3 Department of Computer Science, Brigham Young University, 84606 Provo, USA

**Keywords:** Proteomics, Correspondence, Alignment, Lipidomics

## Abstract

**Background:**

Liquid chromatography-mass spectrometry is a popular technique for high-throughput protein, lipid, and metabolite comparative analysis. Such statistical comparison of millions of data points requires the generation of an inter-run correspondence. Though many techniques for generating this correspondence exist, few if any, address certain well-known run-to-run LC-MS behaviors such as elution order swaps, unbounded retention time swaps, missing data, and significant differences in abundance. Moreover, not all extant correspondence methods leverage the rich discriminating information offered by isotope envelope extraction informed by isotope trace extraction. To date, no attempt has been made to create a formal generalization of extant algorithms for these problems.

**Results:**

By enumerating extant objective functions for these problems, we elucidate discrepancies between known LC-MS data behavior and extant approaches. We propose novel objective functions that more closely model known LC-MS behavior.

**Conclusions:**

Through instantiating the proposed objective functions in the form of novel algorithms, practitioners can more accurately capture the known behavior of isotope traces, isotopic envelopes, and replicate LC-MS data, ultimately providing for improved quantitative accuracy.

## Background

Liquid chromatography-mass spectrometry (LC-MS) is a popular technique for elucidating the composition of liquid samples. Data processing considerations are essential to accurately determine the identity of molecules (analytes such as lipids or peptides) contained in the sample (a process called identification), as well as their quantity in sample (a process called quantification).

Information about sample quantity is captured directly in survey scans, or MS (aka MS1) data. Fragmentation spectra of one or more analytes constitute MS/MS (or MS2) data, and this information is typically used to corroborate or ascertain the identity of a molecule. Partitioning/clustering MS1 signal from complex samples and mapping the signal to other analyses (correspondence) is challenging. Some quantification strategies bypass these challenges by using information derived directly or indirectly from MS/MS data. These methods include spectral counting [1] and isobaric tags for relative and absolute quantification (iTRAQ) [2]. Though these methods have been successful, the amount of quantifiable signal embedded in MS1 data is estimated to far exceed what is currently available by MS/MS [3]; however, most MS1 data remains unused by current software. Hence, improving methods for partitioning and mapping MS1 signal stands to significantly (˜10 fold) increase the sensitivity of a typical label-free or isotope-labeling MS-omics experiment, both for experiments currently being run and for past experiments where raw data is still available.

**Figure 1:**
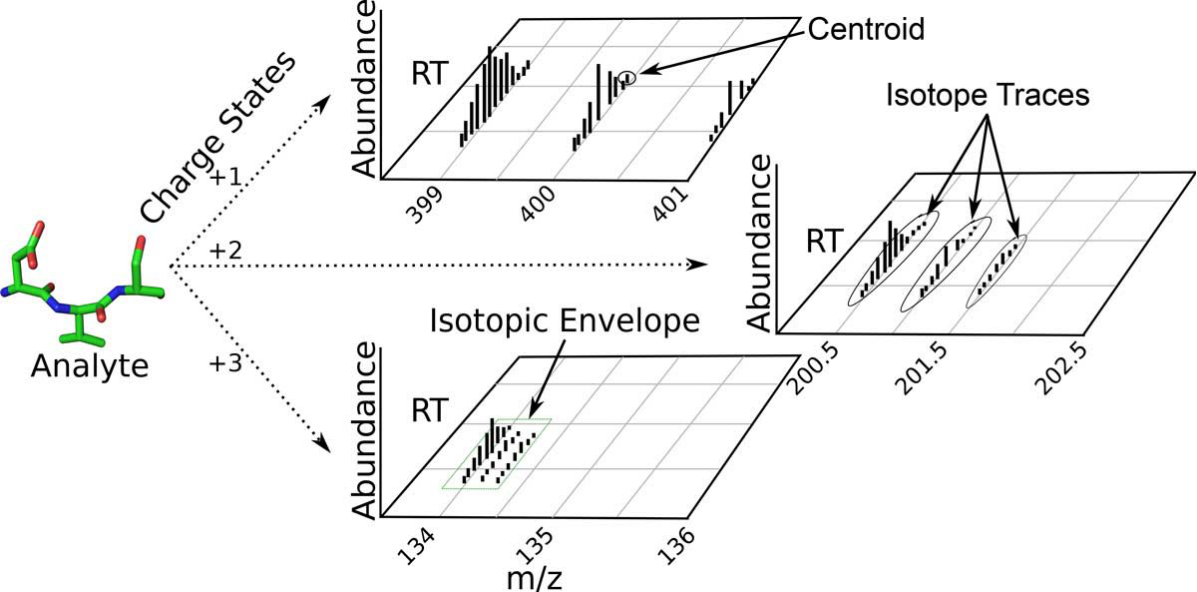
**An LC-MS sample is composed of many instances of many classes of analyte**. Each detected instance of an analyte is ionized to a charge state. The signal produced by each charged analyte is accumulated as a function of the mass of the analyte, its charge (together composing the mass-to-charge ratio (m/z)), and the time at which it is detected (dictated by the chromatographic system in use).

Subdivision of raw mass spectrometer output data into smaller signal partitions attributed to specific analytes in the sample is critical prior to achieving analyte identification and quantification. The larger partition unit, called an isotopic envelope trace, is the signal pattern generated by each analyte/charge combination (see Figure 1). Because mass spectrometers can only detect charged analytes, the sample must be subjected to an ionization method, which imputes a charge on each detected analyte. Since multiple instances of each component exist in the sample, and since each instance is charged independently, there exist in each output the signals of multiple analytes, each with (potentially) multiple charge states. These create a distinct signal--the isotopic envelope trace--for the total signal detected for each analyte/charge state combination. Each isotopic envelope trace is composed of a series of isotope traces, which are manifestations of the fact that each analyte is composed of chemically similar compounds that differ in the weight of certain isotopes (such as ^12^C vs ^13^C). At each charge state, each molecular variant of the analyte is detected at a particular m/z offset, creating one isotope trace per molecular variant/charge-state/analyte combination.

Mass spectrometry data, in its raw form, is not ideal for isotope trace extraction or subsequent processing. After internally accumulating signal over discrete time slices, the mass spectrometer outputs raw data condensed into the form of many narrow profiles wherever signal is present. Conversion to centroid mode integrates the abundance of each of these profiles into a single tuple called a centroid. This is considered a routine conversion for which ample software is readily available. We adopt the typical convention of using centroid data.

Despite the ubiquity of LC-MS experiments, to the best of our knowledge, no concise, complete description of the LC-MS isotope trace and isotopic envelope extraction problems exists. Here, we describe constructs for isotope traces and isotopic envelopes, as well as formally describe the relationship of centroids, isotope traces and isotopic envelopes. In this context, we review extant objective functions for isotope trace extraction, isotopic envelope extraction, and correspondence. Finally, we propose novel objective functions for each of these tasks that address shortcomings in current approaches.

## Results and discussion

### Isotope trace extraction

The most important data processing step in a typical quantitative LC-MS pipeline is isotope trace extraction [4]. Clustering centroids into isotope traces is a non-trivial problem due to the many sources of noise affecting centroid mass and abundance. Sources of noise affecting centroids include chemistry effects due to chromatography, abundance inaccuracy due to ionization efficiencies, m/z deviation due to machine calibration, occlusion/adulteration of low-abundance signal due to dynamic range limitations, and compounded inaccuracies in mass-to-charge ratio (m/z) and abundance due to centroid construction. Of course, these complications are propagated from the clustering of isotope traces to the clustering of isotopic envelopes to the identification of cross-experiment correspondence.

A centroid is denoted as *c *= (*µ, τ, α*) where *µ, τ, α *are values for m/z, retention time (RT), and abundance, respectively. A single MS run produces a set of centroids $C={\left\{c_{i}\right\}}_{i=0}^{n}$, where *n *can readily reach into the millions.

**Figure 2:**
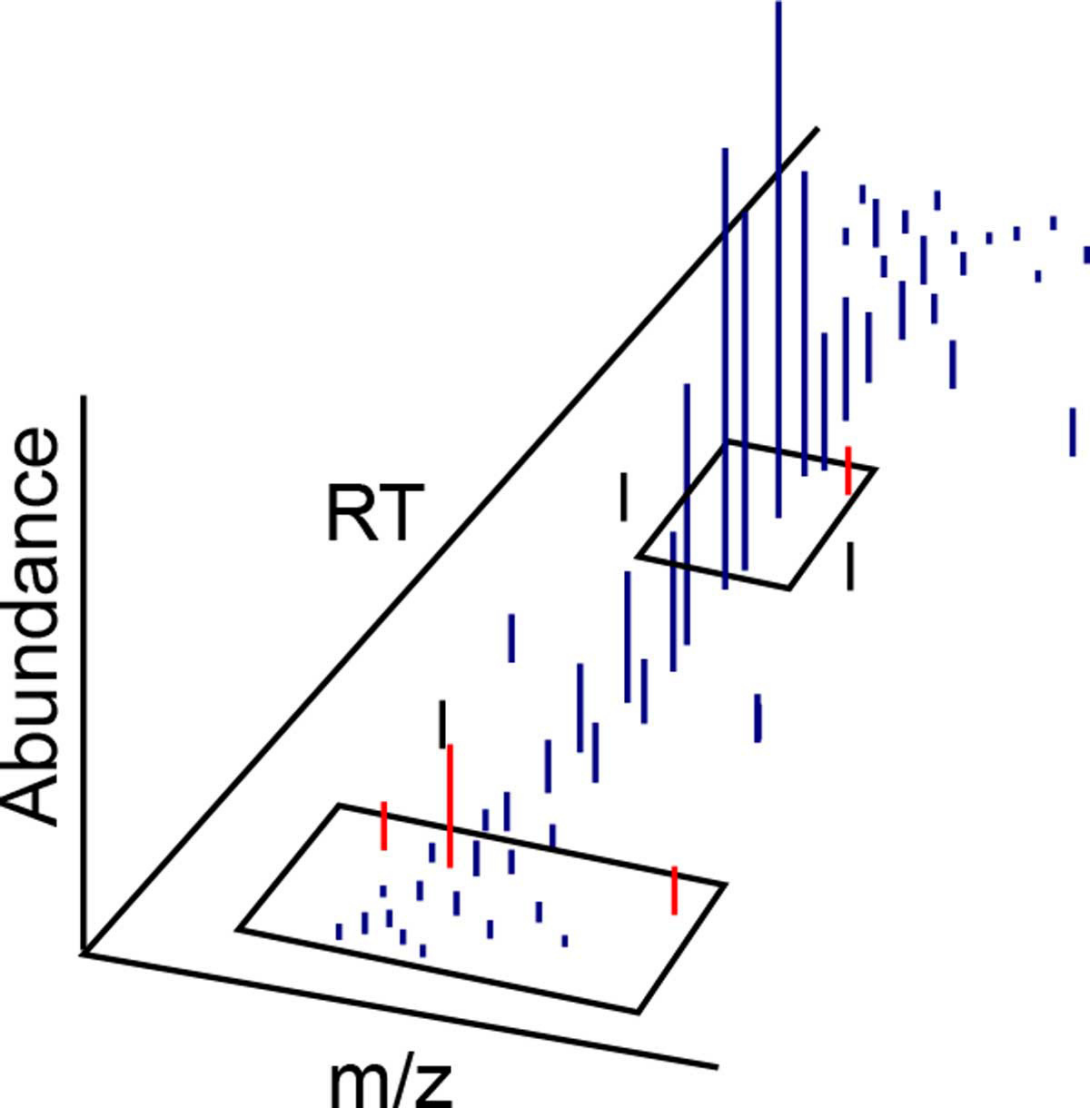
**Each box illustrates an example candidate local neighborhood ϒ defined by an algorithm-specific m/z and RT window**. Blue centroids indicate the centroids pertaining to the isotope trace, while red centroids have been rejected due to differences in abundance, m/z, and/or RT compared to other centroids in ϒ.

**Figure 3:**
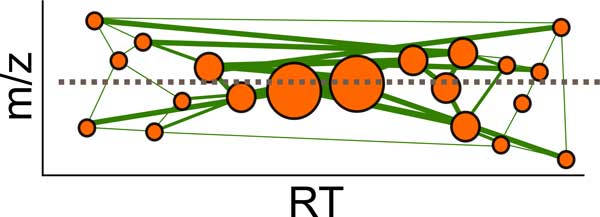
**One way to characterize the relative lack of variance in m/z (compared to RT) of an isotopic trace is by using an inverse variant of the Theil-Sen estimator--a fully-connected graph is constructed with edges connecting each pair of centroids (circles whose radius indicates abundance), and weighted by the abundance of its connected centroids (represented by line thickness)**. An isotopic trace will have a weighted average (inverse) slope of approximately zero (not all connections shown).

An isotope trace *F ⊂ C *is defined as a set of centroids: $F={\left\{c_{i}\right\}}_{i=0}^{m}$, with each set *F *constrained so that all members of a given isotope trace *F *are within a distance threshold *θ *from other centroids in their neighborhood ϒ (see Figure 2):

(1) $$\underset{j\in {ϒ}_{i}}{\text{max}}\;\delta_{F}\left(c_{i},c_{j}\right)<\theta^{\mu ,\alpha ,\tau }$$

where *θ *is a function of centroid m/z, RT, and abundance, *δ_F _*is a distance function based on m/z, RT, and abundance, and ϒ is a neighborhood demarcated by m/z, RT, and abundance. Additionally, the slope of a (abundance-weighted) linear regressor estimate for an isotopic trace is very nearly infinite (in the *m/z, RT*-plane). One way to formalize this is to use a weighted, inverse variant of the Theil-Sen estimator as follows (see Figure 3):

(2) $$\frac{\sum_{c_{i},c_{j}\in F}\frac{c_{j}^{\mu }-c_{i}^{\mu }}{c_{j}^{\tau }-c_{i}^{\tau }}c_{j}^{\alpha }c_{i}^{\alpha }}{\sum_{c_{i},c_{j}\in F}c_{j}^{\alpha }c_{i}^{\alpha }}\approx 0$$

where *c^α ^*is the abundance of centroid *c *and *c^µ ^*is the m/z of centroid *c*.

Note that the behavior of isotope traces are dependent on all three MS dimensions although many common approaches to isotope trace extraction ignore one or more of these dimensions. For example, most proprietary MS software uses hard m/z bins for isotope trace extraction.

#### Extant objective functions

The prominent algorithms for isotope trace extraction include centWave [5], MatchedFilter [5], centroidPicker [6], massifquant [7], and MaxQuant [8].

MatchedFilter operates on the simplifying assumptions that 1) isotope traces are completely contained within pre-processed hard m/z bins and 2) the shapes of all isotope traces in a run can be fit to the same shape. MatchedFilter minimizes the error of a Gaussian fit over prospective isotope traces, by attempting to find the set of isotope traces  F, a scaling factor *b_F _*, and mean retention time *F^t ^*for each isotope trace that minimizes the summed abundance error over all isotope traces. Note the use of a single, global variance *σ*, an average RT width for all *F *∈ F:

(3) $$\lambda_{F}=\underset{F\in F}{\sum }\underset{c\in F}{\sum }\left|bFe^{\frac{-{\left(c^{\tau }-F^{t}\right)}^{2}}{2\sigma^{2}}}-c^{\alpha }\right|$$

The centWave algorithm extracts isotope traces that fit a scaled and translated Ricker wavelet *ζ *(commonly called a Mexican hat function). The fit is calculated as a convolution between the shape function and the signal intensity (abundance), so the goal is to maximize the objective function:

(4) $$\lambda_{F}=\underset{F\in F}{\sum }\underset{c\in F}{\sum }c^{\alpha }\zeta \left(c\right)$$

where

(5) $$\zeta \left(c\right)=\left(\frac{1}{\sqrt{b_{F}}}\frac{2}{\sqrt{3}\pi^{\frac{1}{4}}}\left(1-{\left(\frac{c^{\tau }-t_{F}}{b_{F}}\right)}^{2}\right)e^{\frac{-{\left(\frac{c^{\tau }-t_{F}}{b_{F}}\right)}^{2}}{2}}\right)$$

with isotope trace-specific scaling parameter *bF *and translation parameter *t_F _*chosen to maximize the convolutional fit over isotope trace *F *.

The algorithm centroidPicker uses heuristic operations on a neighborhood graph to separated the data into connected components. It connects an undirected graph *G *= (*C, N*) of centroids where the edges *N *are constrained such that:

(6) $$N=\left\{\left(c_{i},c_{j}\right)\left|\begin{array}{c} \delta_{c}\left(c_{i},c_{j}\right)<\delta_{c}\left(c_{i},c_{k}\right)\forall_{k\neq j}\\ c_{i}^{\alpha }>\theta \text{ and }c_{i}^{\alpha }>\theta \end{array}\right)\right\}$$

for some intensity threshold *θ *and centroid distance function *δ_c_*, resulting in *G *being composed of one or more connected components, each considered one isotope trace. Thus, $F=\left\{F_{i}|\forall c_{k}\in F_{i},\exists_{cl\in F_{i}}\left\{c_{l}\in ϒ\left(c_{k}\right)\right\}\right\}$, where the neighborhood function ϒ (*c*) returns the set of nodes connected to *c *(and is symmetric because *G *is undirected).

The objective functions for massifquant and MaxQuant define  F as the set of all *F *formed by iterating over values of time *t*, and adding *c *if *c^τ ^*= *t *and $\left|c^{\mu }-c_{*}^{\mu }\right|<\in$, where *c*_∗ _∈ *F *and $c^{\tau }-c_{*}^{\tau }\leq c^{\tau }-c_{j}^{\tau }$ for all *c_j _*∈ *F*. For massifquant, ∈ is prescribed by a Kalman filter induced from the variance in *c^µ ^*and *c^α ^*for all *c_j _*∈ *F *such that $c_{j}^{\tau }<t$, with the added constraint that *c^τ ^*be unique in *F *. MaxQuant defines ∈ simply as a distance threshold of 7 ppm m/z.

#### Proposed objective functions

We define *F^µ^*, the m/z of isotope trace *F*, given by the weighted m/z of its component centroids:

(7) $$F^{\mu }=\frac{\underset{c\in F}{\sum }c^{\alpha }c^{\mu }}{\underset{c\in F}{\sum }c^{\alpha }}$$

and using it propose an alternative objective function for isotope trace extraction:

(8) $$\lambda_{F}=\underset{F\in F}{\sum }\underset{c\in F}{\sum }\left|b_{F}\left(\tau \right)e^{\frac{-{\left(c^{\tau }-F^{t}\right)}^{2}}{2\sigma_{F}^{2}}}a_{F}\left(\alpha \right)e^{\frac{-{\left(c^{\mu }-F^{\mu }\right)}^{2}}{2h{\left(\alpha \right)}^{2}}}-c^{\alpha }\right|$$

where, again, centroid clustering  F and retention time means *F^t ^*are chosen to minimize the Gaussian fit error; however, rather than using a single global variance in the RT dimension, each isotope trace *F *has a local variance *σ_F_*; in addition, the scaling factors have become time-dependent scalar functions *b_F_*(·). The second Gaussian factor, parameterized by mean *F^µ ^*and variance function *h*(·), models the m/z width of the isotope trace, which is a function of the abundance *α*. Isotope traces splay at low abundance and narrow at high abundance; thus, both the variance *h*(·) and the scaling factors *a_F_*(·) are modeled as functions dependent on the abundance *α*. Note that while variance is trace-independent (depending only on abundance), each isotope trace has its own scaling function (which in turn is dependent on abundance).

#### Alleviating current limitations in isotopic trace extraction

Current objective functions for isotopic trace extraction fail to capture isotopic trace behavior formalized in this section: namely, a pattern of centroids forming a generally tight distribution through time around a specific m/z, with variation occurring as a factor of abundance, with normal abundance traces splaying at the beginning and end of elution, and lower abundance traces displaying high m/z variance in general. Moreover, isotope traces are skewed in time, with sharp onset of intensity followed by a post-peak long tail. The shape of traces is almost never strictly Gaussian (or even symmetric), as chromatography almost always deviates from the Gaussian in heading (which is more steep) and in tailing (which is less steep). Our objective functions account for each of these behaviors.

### Isotopic envelope extraction

The LC-MS clustering problem is defined as a two-step partitioning problem. In the first step, isotope trace extraction, we require a partition *ϕ *of the set of all centroids *C *into the set of isotope traces  F, $\phi \left(C\right)={\left\{F_{i}\right\}}_{i=1}^{r}=F$with the properties:

(9) $$\overset{r}{\underset{i=1}{\cup }}F_{i}=C\;\text{and}\;F_{i}\cap F_{j}=\emptyset \;\forall_{F_{i}\neq F_{j}\in F}$$

In other words, 1) all centroids are assigned to an isotope trace; 2) isotope traces can't share centroids. Because any sensor's detection of a physical system will deviate somewhat from the true physical system, we can expect MS detections to contain extraneous centroids. However, all signal ought to be accounted for (even if some identified "traces" eventually are identified as noise) and, in a platonic model, ought to be assigned to an isotope trace.

In the second step, isotopic envelope extraction, we require a partition *ψ *of the set of isotope traces  F into the set of isotopic envelopes $\varepsilon ,\;\psi \left(F\right)={\left\{E_{i}\right\}}_{i=1}^{p}=\varepsilon$with the property

(10) $$\overset{p}{\underset{i=1}{\cup }}E_{i}=F$$

The choice of partitions *φ *and *ψ *is guided by a set of distance functions Δ that define distances between centroids, isotope traces, isotopic envelopes, etc. and objective functions *λ_F _*and *λ_E _*that describe "good" isotope traces and isotopic envelopes, respectively. The choice of distance and objective functions, along with choice of optimization procedure, characterizes an algorithmic approach for solving this clustering problem. A defining general property of isotopic envelopes, however, is the regular spacing between component isotope traces. In addition, for virtually all molecules from biological sources we expect that if there is an isotope with index *j *and an isotope with index *j *+ 2, then there exists an isotope with index *j *+ 1.

An isotopic envelope *E *is the set of isotope traces *F_i _*that are produced by a given analyte/charge state combination: $E={\left\{F_{i}\right\}}_{i=0}^{q}$ subject to the constraint that the m/z difference between each consecutive (assuming an ordering of centroids from least mass to greatest mass) isotope trace in *E *must be equivalent to $\frac{k}{z_{E}}+\in$, where *k *is the mass of a neutron, *z_E _*is the integer charge of *E *and ∈ is a noise tolerance parameter. That is, assuming an indexing function $\iota^{µ}:\varepsilon \times N\to F$ that returns the *i*th least massive isotope trace in an isotopic envelope:

(11) $$l^{\mu }\left(F,i+1\right)=l^{\mu }\left(F,i\right)=\frac{k}{z_{E}}+\in ,\;1\leq i\leq |E|-1$$

The m/z *m *of the *j*th isotope trace in *E *must be roughly equivalent to

(12) $$m=\frac{\tilde{m}+jk}{z}$$

where $\tilde{m}$ is the uncharged molecular weight of the ion.

Every isotope trace consists of signal from at least one isotopic envelope, and, in the case of overlapping isotopic envelopes, an isotope trace may be composed of signal from more than one isotopic envelope.

#### Extant objective functions

FeatureFinder [9] is an isotopic envelope extraction algorithm in OpenMS that searches directly for *E*. Although the details are not completely clear, it appears that the algorithm attempts to minimize

(13) $$\lambda_{E}=\underset{E\in \varepsilon }{\sum }\underset{c\in E}{\sum }G_{E}\left(c\right)$$

where the *G_E _*compute a comparison between the (*µ*, *τ*, *α*) values for a centroid and the expected centroid values obtained from a heuristic isotopic envelope shape. Note that isotopic trace extraction is ignored.

MSInspect [10], another approach to isotopic envelope extraction, groups all coeluting signals and compares them to a simulated envelope calculated from a Poisson distribution parameterized by m/z, with the goal being to minimize the KL divergence between the Poisson distribution and the "distribution" of abundance in an instantaneous profile of the envelope at time *τ *:

(14) $$\lambda_{E}=\underset{F\in E,c\in^{\tau }F}{\sum }\hat{P}\left(c^{\alpha }\right)\text{log}\frac{\hat{P}\left(c^{\alpha }\right)}{P_{m}\left(c^{\mu }\right)}$$

where the notation *c *∈*^τ ^F *means that *c *∈ *F *at time *τ*, *E *is the maximal intensity (instantaneous) isotopic envelope (at time *τ*), $\hat{P}\left(\cdot \right)$ is the ratio of the intensity of isotope trace *F *(at time *τ*) to the total intensity of all isotope traces *F *∈ *E *(at time *τ*), and *P_m_*(·) is the value of the Poisson distribution at *c^µ^*.

#### Proposed objective functions

We propose an alternative objective function for isotopic envelope extraction:

(15) $$\lambda_{E}=\beta I\left(E\right)+\left(1-\beta \right)J\left(E\right),0\leq \beta \leq 1$$

where *β *is a relative importance weighting coefficient. The first term computes the deviation of member isotope traces from the expected charge-based m/z interval--we want the isotope traces in envelope *E *to fit expected m/z spacing:

(16) $$I\left(E\right)=\underset{\begin{array}{c} F_{i},F_{j}\in E\wedge \\ F_{i}^{\mu }<F_{j}^{\mu }\wedge \\ \forall_{F_{k}^{\mu }\in E}F_{k}^{\mu }>F_{i}^{\mu }\Rightarrow F_{k}^{\mu }>F_{j}^{\mu }\vee F_{k}=F_{j} \end{array}}{\sum }|\left(F_{i}^{\mu }-F_{j}^{\mu }\right)-\frac{k}{z_{E}}|$$

The second term computes the deviation in elution time of member isotope traces--we want all the isotope traces in isotopic envelope *E *to co-elute within a small time window:

(17) $$J\left(E\right)=\underset{F_{i},F_{j}\in E}{\sum }F_{i}^{\tau }-F_{j}^{\tau }$$

where *F^τ ^*could be defined analogously to Equation 7, could be the maximum intensity for isotopic trace *F *or could be some other reasonable definition for isotopic trace elution time.

We want to optimize *ε *and the *z_E _*so that *λ_E _*is minimized; that is, we want to find charge-state/isotopic-envelope pairs such that the errors in expected m/z and co-elution time are minimized.

The isotopic envelope extraction segment of the MaxQuant [8] algorithm is one of the possible instantiations of this objective function, though many possibilities exist for how to set the allowable m/z and RT error and how to generate the prerequisite list of isotope traces.

#### Alleviating current limitations in isotopic envelope extraction

Isotopic envelopes are rich with data: the expectation of contiguous isotope traces with a uniform m/z charge gap, and similar maximal abundance across all isotope traces. Accounting for this behavior is not possible without adopting an isotope trace-centric approach to data extraction. Reliance upon maximal elution time alone--an approach that is susceptible to conflation with overlapping envelopes in complex samples--is not a sensitive approach in envelopes of lower abundance, where maximal elution times are not pronounced. Moreover, by first finding the isotope traces, the exact m/z of each isotope trace can be calculated using a weighted average, alleviating the need for larger than theoretically justified isotope trace gaps, which will not be sensitive in complex samples with overlapping isotopic envelopes. Instead, the proposed objective functions leverage a precise and reliable m/z charge gap and adjacency of isotope traces along with maximal elution times, using all the information in the data.

### Correspondence

**Figure 4:**
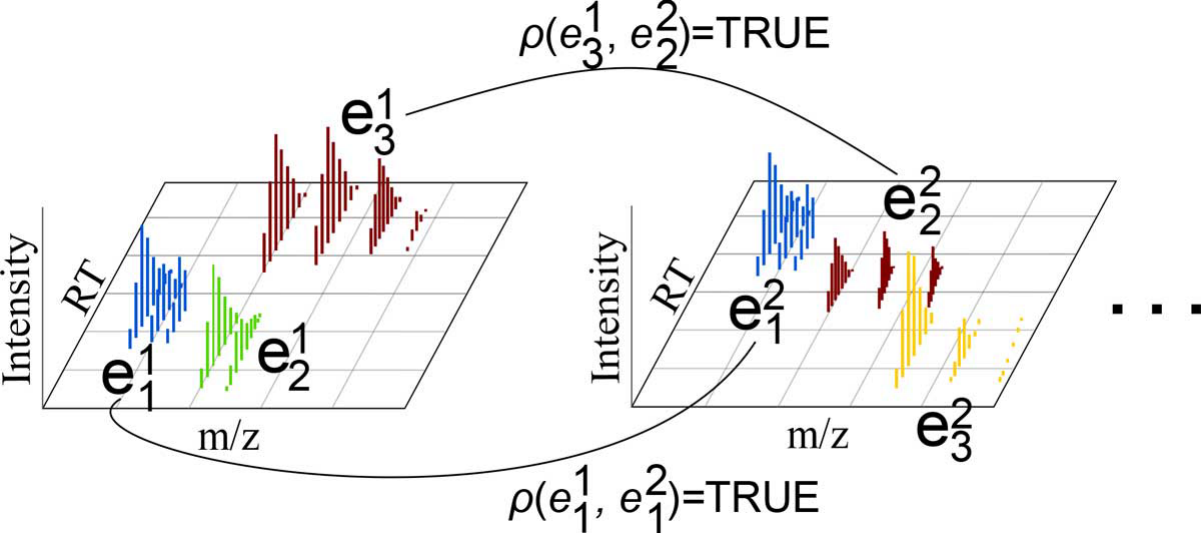
**Objective functions for correspondence must allow a mapping from an isotopic envelope in one run to an envelope in another, or to none, if there is no corresponding isotopic envelope**. Here, the unillustrated relations would yield FALSE.

The final objective of almost every MS experiment is the differential analysis of more than one MS run. This comparison allows the identification of significant quantity and component differences, useful for applications such as drug design, disease treatment, biological processes research and chemical forensics. Correspondence yields a mapping between isotopic envelopes in different runs (see Figure 4), a prerequisite for differential analysis.

The combination of noise from within one run (enumerated above) and noise from run to run--most notable in retention time shifts, where an isotopic envelope appears at a different retention time or with a compressed or stretched RT length compared to another run--make LC-MS correspondence non-trivial.

The correspondence mapping should again optimize an objective function which, in turn, characterizes an algorithm choice for solving the correspondence problem.

#### Extant objective functions

According to a recent review on LC-MS correspondence algorithms [11], all extant approaches use either centroid data or a reduction of isotopic envelope traces into a single centroid. Of the almost sixty algorithms reviewed there, nearly all use the same objective function--finding a family of one-to-one partial functions χ*_r _*: ε*_r _→ *ε*_∗ _*(a different function for each experimental run *r*), where ε*_∗ _*is the set of envelopes from a reference run, that minimizes global RT and m/z distance between isotopic envelopes (in any of their reduced forms, according to the authors):

(18) $$\lambda_{corr}=\underset{E\in \varepsilon_{r}}{\sum }\delta {\left(E,\chi_{r}\left(E\right)\right)}^{\tau ,\mu }$$

where *δ*()*^τ,µ ^*is a distance function defined over RT and m/z.

The continuous profile model (CPM) [12] uses a different objective function, and thus is free from the reference requirement that most other algorithms have, allowing for a symmetric solution (one that is not dependent on the choice of a reference run). Additionally, the mapping is somewhat more localized than that of most correspondence algorithms. CPM minimizes the log likelihood of differences between a hidden Markov model *mτ *of the RT of a latent run and observed runs:

(19) $$\lambda_{corr}=\text{log}p\left(D|m^{\tau }\right)$$

where *D *is the set of observed runs.

#### Proposed objective functions

In contrast to existing LC-MS correspondence objective functions, the objective functions suggested here use the entire isotopic envelope. This allows greater discrimination by using isotope trace quantity and spacing to match isotopic envelopes from different runs. This extra discrimination is essential given the amount of RT variance and (to a lesser degree) m/z variance present in the data.

Let *R *be a set of runs, each of which has an associated set of isotopic envelopes $\varepsilon_{r}={\left\{E_{i}^{r}\right\}}_{i=1}^{pr},1\leq r\leq |R|$ and let $\tilde{\varepsilon }=\cup_{r}\varepsilon_{r}$. We seek to find a binary equivalence relation *ρ *that induces a set of *correspondence classes *over $\tilde{\varepsilon }$ that is reflexive (an envelope corresponds with itself), symmetric (if envelope *E*_1 _from run 1 corresponds with envelop *E*_2 _from run 2, then *E*_2 _also corresponds with *E*_1_) and transitive (if envelope *E*_1 _from run 1 corresponds with envelope *E*_2 _from run 2 and envelope *E*_2 _corresponds with envelope *E*_3 _from run 3, then *E*_1 _corresponds with *E*_3_); and if $\rho \left(E_{i}^{r},E_{j}^{s}\right)=\text{TRUE}$, then for *k *≠ *i*, $\rho \left(E_{k}^{r},E_{j}^{s}\right)=\text{FALSE}$ and for *k *≠ *j*, $\rho \left(E_{i}^{r},E_{k}^{s}\right)=\text{FALSE}$ (an envelope from one run may have 0 or 1 matches from any other run; note that due to reflexivity, this also means that two non-identical envelopes from the same run never correspond).

This relation should minimize

• The difference in charge state between corresponding isotopic envelopes, $\delta_{charge}$.

• The difference in m/z between isotope traces in corresponding isotopic envelopes, $\delta_{mz_{it}}$.

• The difference in elution duration between isotope traces in corresponding isotopic envelopes, $\delta_{dur}$.

• The difference in isotope abundance ratios between corresponding isotopic envelopes, $\delta_{ratio}$.

• The difference in m/z between corresponding isotopic envelopes, $\delta_{mz_{ie}}$.

• The number of singleton correspondence classes, $\delta_{orphan}$.

• The difference in retention time between corresponding isotopic envelopes, $\delta_{rt}$.

An objective function incorporating all of these variables can take many forms, with perhaps the simplest generalization being a weighted linear combination, with weighting coefficients *ω *allowing relative prioritization:

(20) $$\begin{array}{c} \lambda_{corr}=\underset{\rho \left(E_{1},E_{2}\right)}{\sum }\omega_{charge}\delta_{charge}\left(E_{1},E_{2}\right)+\omega_{mz_{it}}\delta_{mz_{it}}\left(E_{1},E_{2}\right)\\ +\omega_{dur}\delta_{dur}\left(E_{1},E_{2}\right)+\omega_{ratio}\delta_{ratio}\left(E_{1},E_{2}\right)\\ +\omega_{mz_{ie}}\delta_{mz_{ie}}\left(E_{1},E_{2}\right)+\omega_{orphan}\delta_{orphan}\left(E_{1},E_{2}\right)\\ +\omega_{rt}\delta_{rt}\left(E_{1},E_{2}\right) \end{array}$$

with the summation over *ρ*(*E*_1_, *E*_2_) meaning a summation taken over all pairs of envelopes *E*_1_, $E_{2}\in \tilde{\varepsilon }$ for which *ρ*(*E*_1_, *E*_2_) = TRUE. Given the weighting coefficients *ω*, the most desirable correspondence would be that induced by the relation ρ* that minimizes *λ_corr _*(see Figure 4),

$$\rho *=\underset{\rho }{\text{argmin}{\;\lambda }_{corr}}$$

#### Alleviating current limitations in correspondence

Recently, several ubiquitous shortcomings were identified in a review of over 50 LCMS correspondence algorithms [11]. The most significant of these shortcomings was the fact that all current LC-MS correspondence algorithms make model assumptions that fail to capture common behavior. In other words, each algorithm is constructed in such a way that the algorithm is guaranteed to get the wrong answer under certain conditions that are common to real LC-MS data. The behaviors discussed included the ideas that:

• Not all analytes appear in all replicates.

• Elution order can swap.

• Shifts occur in m/z as well as in RT.

Some correspondence methods reduce isotopic envelopes to a single point representation. This deprives the method of a rich source of distinguishing data found in full isotopic envelopes--the expectation of contiguous isotope traces with a uniform m/z charge gap, number of isotope traces, and relative abundance ratio of isotope traces. Similarly, most correspondence algorithms conduct an initial RT alignment, where signals (almost always much-reduced from the full isotopic envelope, and rarely built up from isotope traces to isotopic envelopes) are shifted up or down in RT (preserving original order) in order to most closely match a reference run. This is invariably followed by direct matching. The problem is that the initial warping is a lossy procedure that adulterates the original RT time, which would be useful to probabilistically ascertaining the closest corresponding isotopic envelope.

The proposed objective function does not force matches between runs, as it is very common for species to either not be present or fall below the signal-to-noise ratio in differential studies. Instead, the proposed objective function leverages the full breadth of isotope envelope information, allowing a rigorous direct comparison of candidate correspondences based on all available data to select the most likely correspondence (in the sense of minimizing error), or no correspondence at all if that is the most likely case given the data.

## Conclusions

We present a concise attempt to formalize LC-MS data clustering problems, describing the constructs of isotope traces and isotopic envelopes and their relational structure. We provide a review of current approaches to isotope trace extraction and LC-MS correspondence, and propose novel objective functions for both tasks that address shortcomings in current methods.

## Competing interests and declarations

The authors declare that they have no competing interests. The publication costs for this article were funded by the University of Montana Office of Research and Sponsored Programs.

This article has been published as part of *BMC Bioinformatics *Volume 16 Supplement 7, 2015: Selected articles from The 11th Annual Biotechnology and Bioinformatics Symposium (BIOT-2014): Bioinformatics. The full contents of the supplement are available online at http://www.biomedcentral.com/bmcbioinformatics/supplements/16/S7.

## Authors' contributions

RS, JTP and DV all contributed in writing this manuscript.
